# Expression of the proteolysis-inducing factor core peptide mRNA is upregulated in both tumour and adjacent normal tissue in gastro-oesophageal malignancy

**DOI:** 10.1038/sj.bjc.6602989

**Published:** 2006-02-21

**Authors:** D A C Deans, S J Wigmore, H Gilmour, M J Tisdale, K C H Fearon, J A Ross

**Affiliations:** 1 Department of Clinical and Surgical Sciences, Cell Injury and Apoptosis Section, Tissue Injury and Repair Group, MRC Centre for Inflammation Research, Edinburgh University, The Chancellor's Building, (SU227) 49 Little France Crescent, Edinburgh EH16 4SB, UK; 2 Department of Pathology, Royal Infirmary, 51 Little France Crescent, Old Dalkeith Road, Edinburgh EH16 4SA, UK; 3 Department of Pharmaceutical Sciences, Aston University, Birmingham, UK

**Keywords:** real-time PCR, cachexia, inflammation, prognosis

## Abstract

Gastro-oesophageal cancer is associated with a high incidence of cachexia. Proteolysis-inducing factor (PIF) has been identified as a possible cachectic factor and studies suggest that PIF is produced exclusively by tumour cells. We investigated PIF core peptide (PIF-CP) mRNA expression in tumour and benign tissue from patients with gastro-oesophageal cancer and in gastro-oesophageal biopsies for healthy volunteers. Tumour tissue and adjacent benign tissue were collected from patients with gastric and oesophageal cancer (*n*=46) and from benign tissue only in healthy controls (*n*=11). Expression of PIF-CP mRNA was quantified by real-time PCR. Clinical and pathological information along with nutritional status was collected prospectively. In the cancer patients, PIF-CP mRNA was detected in 27 (59%) tumour samples and 31 (67%) adjacent benign tissue samples. Four (36%) gastro-oesophageal biopsies from healthy controls also expressed PIF-CP mRNA. Expression was higher in tumour tissue (*P*=0.031) and benign tissue (*P*=0.022) from cancer patients compared with healthy controls. In the cancer patients, tumour and adjacent benign tissue PIF-CP mRNA concentrations were correlated with each other (*P*<0.0001, *r*=0.73) but did not correlate with weight loss or prognosis. Although PIF-CP mRNA expression is upregulated in both tumour and adjacent normal tissue in gastro-oesophageal malignancy, expression does not relate to prognosis or cachexia. Post-translational modification of PIF may be a key step in determining the biological role of PIF in the patient with advanced cancer and cachexia.

Although cachexia is a major cause of morbidity and mortality among cancer patients, the mechanisms remain unclear. Proteolysis-inducing factor (PIF) has been identified as a possible cachectic factor (Todorov *et al*, 1996). Initially isolated from a murine tumour model (MAC16), a human homologue of PIF was subsequently identified in human urine from weight losing cancer patients (Todorov *et al*, 1996; Cariuk *et al*, 1997; Wigmore *et al*, 2000). Proteolysis-inducing factor has been named as such because it has been shown to induce skeletal muscle proteolysis both *in vitro* and *in vivo* (Lorite *et al*, 1997, 2001; Watchorn *et al*, 2001). The molecule demonstrates a high degree of glycosylation, consisting of a peptide core of 4 kDa and carbohydrate residues contributing to the estimated total molecular size of 24 kDa (Todorov *et al*, 1997). The glycosylation appears essential for its proteolytic activities, as neither the peptide core alone nor de-glycosylated native PIF has any effect on weight loss in mice (Todorov *et al*, 1996).

In hepatocytes, glycosylated PIF has been shown to stimulate production of the cytokines interleukin-6 (IL-6) and IL-8 and the acute-phase protein C-reactive protein (CRP) via induction of transcription factors NF-κB and STAT3 (Watchorn *et al*, 2001). Therefore, PIF may contribute to the inflammatory state observed in conjunction with cancer cachexia in addition to its proteolytic function, and could conceivably also play a role in the generation of a proinflammatory state outwith the context of cancer.

Some gastrointestinal cancers and cancer cell lines have been shown to produce glycosylated PIF protein (Cabal-Manzano *et al*, 2001). Glycosylated PIF has been detected within tumour cells by immunohistochemistry and in the urine of the same subjects by Western blot. The presence of glycosylated PIF in tumour cells was also associated with increased weight loss among these patients. Proteolysis-inducing factor has also been identified from the human melanoma cell line (G361) (Todorov *et al*, 1999) and the pancreatic cell line (MIA PaCa2) (unpublished data).

Human-derived PIF has been designated human cachexia-associated protein (HCAP) by early identification of the human gene sequence (GenBank accession number AR053250) and patent (US 5834192) (Akerblom and Murry, 1998) and later by a group, which investigated its expression in prostate cancer patients (Wang *et al*, 2003). mRNA of HCAP was detected in 13 out of 15 radical prostatectomy specimens and seven out of nine bone metastases, but was not detectable in normal prostate or in any adjacent non-malignant prostate tissue from prostatectomy samples. *In situ* hybridisation confirmed that HCAP mRNA was only detectable in cancer cells and not in surrounding stromal cells or benign prostate tissue. Expression of HCAP mRNA was also detected from prostate cancer cell lines. When two of these cell lines (PC-3M and LuCaP35) were implanted into mice, there was a strong correlation between HCAP mRNA expression from the cancer cells and weight loss among the mice (Wang *et al*, 2003).

A BLAST search of the human genome using the PIF core peptide (PIF-CP) cDNA sequence (GenBank accession number AY590150) identified three products that arise from the single gene locus on 12q3.1; dermcidin (DCD), neuronal survival-promoting peptide, and a candidate breast cancer oncogene. Dermcidin is a novel peptide that was identified from human sweat and was shown to possess antimicrobial properties (Schittek *et al*, 2001). Unlike the PIF molecule, DCD appears to be unglycosylated. Dermcidin mRNA was detected in normal skin, benign naevi, and malignant melanoma cells by RT–PCR. Screening of adult and fetal tissue panels did not identify DCD mRNA in any other tissue. The authors therefore concluded that DCD was exclusive to skin and associated appendages.

Another PIF homologue has been identified following its isolation from the culture medium of neuronal cells grown under oxidative stress (Cunninghan *et al*, 1998). *In vivo* studies suggest that this protein confers survival benefits to hypoxic neuronal cells and the molecule has been termed neuronal diffusible survival-promoting peptide.

The third peptide, which has been mapped to the PIF gene locus, was identified using SAGE in breast cancer tissue (Porter *et al*, 2003). The gene was expressed only in a subset of invasive breast carcinomas and their lymph node metastases. *In situ* hybridisation studies located the mRNA to cancer cells only with no expression found within stromal cells. Northern blot analysis of 75 human adult and fetal tissues detected RNA expression only in the pons and paracentral gyrus of the brain. The protein was identified in 10% of invasive breast cancers, two out of 64 pancreatic cancers, and normal sweat glands. Receptors were found in the brain and on tumour cells that were producing the protein, suggesting an autocrine/paracrine mode of action. When the cDNA sequence was introduced into a breast cancer cell line (21NT), these cells had accelerated growth and were more resistant to oxidative stress and hypoglycaemia. The authors have suggested that this gene may function as an oncogene in breast cancer with survival-promoting properties.

More recently, a group of investigators probed a panel of normal human tissue cDNA for PIF-CP using real-time PCR and found absent or very low levels of expression (Monitto *et al*, 2004). In an analysis of unpaired breast tissue samples, minimal PIF expression in normal breast tissue was detected. However, significantly elevated PIF expression within breast tumours was observed. Mice implanted with tumours transfected with a PIF vector were found to produce increased levels of PIF mRNA and protein but the protein was not glycosylated and these mice did not develop wasting. However, the tumours from these mice were significantly larger than controls, supporting a previous hypothesis that PIF may confer cell survival properties (Cunningham *et al*, 1998).

PIF mRNA and protein are not expressed in most normal tissues and are overexpressed by certain tumours. We investigated the expression of the PIF-CP gene in paired tissue samples from patients with gastric or oesophageal tumours and compared levels of expression between tumour tissue and adjacent non-neoplastic tissue using the PIF mRNA sequence that our group had previously cloned (GenBank accession number AY590150). We also compared levels of gene expression with nutritional status and investigated the possible survival-promoting role of the PIF-CP gene on prognosis and survival among the patient cohort.

## MATERIALS AND METHODS

### Study patients

All patients diagnosed with gastric or oesophageal cancer between June 2002 and March 2004 within Lothian and Borders regions were invited to take part in the study. Patients who were not suitable for surgical resection were excluded. Patients provided written informed consent and the study received ethical permission from the Lothian Research Ethics Committee. Clinical information was collected prospectively. Patients were staged according to the International Union Against Cancer (UICC) (Sobin and Wittekind, 2003), and final histopathological stage (pTNM) was used in all cases. Tumours around the oesophago-gastric junction were classified according to Siewert and those classified as type I and II were classified as oesophageal tumours, and type III as gastric cancers (Siewert and Stein, 1998). Full clinical and pathological information was recorded for each patient, including treatment modality. Patients provided urine samples at the time of recruitment and whole blood was collected at the same time. Duration of survival, defined as time from histological diagnosis to death, was recorded for all patients and all deaths were disease related.

Gastric or oesophageal biopsies were collected from 11 healthy volunteers at the time of endoscopic examination. These patients were undergoing endoscopic investigation of gastro-intestinal symptoms. In all instances, both the macroscopic and microscopic assessments were considered normal. Those patients with abnormal findings were excluded as healthy controls. The tissue was sampled with biopsy forceps and the histological appearances were confirmed as normal by a consultant pathologist (HG).

### Nutritional assessment

Nutritional assessment including calculation of body mass index from the patients’ weight and height and measurement of mid-arm circumference (MAC) and triceps skinfold thickness (triceps) was performed as described previously (Wigmore *et al*, 2000). Mid-arm muscle circumference (AMC) was calculated by means of Jelliffe's (1966)equation. Premorbid patient weight was recalled by the patient and confirmed where possible from the medical notes. Anthropometry measurements were normalised using standardised reference tables (Bishop *et al*, 1981).

### Tissue collection

Tissue was obtained from patients at the time of surgical resection. A section of tumour tissue and a sample of benign mucosa from the same organ were collected from each patient. A single consultant pathologist (HG) analysed tissue sections to confirm the presence of malignant cells in the tumour samples and the absence of malignant cells within the benign samples. Tissue samples were snap-frozen in liquid nitrogen before storage at −80°C until further analysis. The average time from tissue collection to freezing was 15 min (range 10–25 min). Tumour tissue and benign tissue were collected from the same patient in all cases. Tissue biopsies collected from healthy controls were snap-frozen within 1 min of collection.

### Quantitative polymerase chain reaction (Q-RT–PCR)

#### RNA isolation and reverse transcription

Total RNA was isolated from tissue samples using the RNAeasy kit (Qiagen Inc., Crawley, UK). RNA quality and integrity was assessed using an Agilent 2100 bioanalyser (Agilent Technologies Ltd, Chesire, UK) in five samples. For the remaining samples, purity and concentration were determined using spectrophotometry (Ultrospec 2000, Pharmacia Biotech, Bucks, UK). RNA samples were treated with DNase (Qiagen Inc., UK) and all RNA samples were checked for genomic DNA contamination before reverse transcription using standard PCR for cytochrome *b*.

Reverse transcription was performed once DNA contamination had been excluded. The reaction mixture included RNA (1 *μ*g in 10 *μ*l diethyl-pyrocarbonate (DEPC)-treated water), 4 *μ*l MgCl_2_ (25 mM), 2 *μ*l 10 × reverse transcriptase buffer, 2 *μ*l dNTPs (10 mM), 1 *μ*l random hexamers (500 *μ*g ml^−1^), 1.5 *μ*l AMV reverse transcriptase (10 U *μ*l^−1^), and 0.5 *μ*l recombinant RNase inhibitor (40 U *μ*l^−1^) (all reagents Promega, Southampton, UK). Reverse transcription was performed at 42°C for 60 min followed by 95°C for 5 min.

#### Real-time PCR

Quantitative PCR was performed using the ABI PRISM 770 real-time Sequence Detection System (Applied Biosystems, Warrington, UK). Reactions were performed in 50 *μ*l total volume, consisting of 25 *μ*l Taqman universal PCR master-mix (UNG × 2), 14 *μ*l primer/probe mix, 2.5 *μ*l ribosomal 18S primer/probe mix (all reagents Applied Biosystems, Warrington, UK), 3.5 *μ*l DEPC-treated water, and 5 *μ*l cDNA. The reaction conditions were 2 min at 50°C, 10 min at 95°C, and 40 cycles with 15 s at 95°C and 1 min at 60°C. The primers and probes were designed using Primer Express® software based on our previously registered sequence for PIF (GenBank accession number AY590150) and the sequences were checked for compatibility by Applied Biosystems (Warrington, UK). The primers were 5′-CAAAAGGAAAATGCAGGTGAAGA, 5′-TGGAAAAAGGCCTAGACGGAG, and the probe 5′FAM-ACAGGCACCAAAGCCAAGGAAGCA-TAMRA.

Quantification of gene expression was calculated using the comparative (ΔΔ*C*_T_) method, where samples were compared with the positive control (Bustin, 2000). Human ribosomal 18S was used as the internal control for all PCR reactions. The values generated for the gene of interest (PIF-CP) were normalised to the internal control as follows: the average threshold cycle numbers for PIF (*C*_T_ FAM) and for 18S (*C*_T_ VIC) were calculated for each sample and the mean VIC *C*_T_ values were subtracted from the mean FAM *C*_T_ values (FAM C_T_−VIC *C*_T_=Δ*C*_T_). Samples that generated cycle numbers greater than 23 for the internal control (18S; VIC) were considered too dilute for accurate analysis and these samples were repeated.

The level of gene expression within each sample was then expressed as a percentage of the total level of gene expression by the positive control (MIA PaCa2). Expression of PIF-CP by the positive control was also initially normalised to the internal control. Then, ΔΔ*C*_T_ was calculated by subtracting the Δ*C*_T_ value of the positive control sample from the Δ*C*_T_ value for each sample (i.e. Δ*C*_T_ sample−Δ*C*_T_ control=ΔΔ*C*_T_). The relative level of expression, normalised to the endogenous control and relative to the positive control, was then calculated by the formula 2^−ΔΔ*C*_T_^. The level of gene expression of the positive control was assigned an arbitrary expression value of 1 and levels of gene expression in the samples were expressed as percentages of the positive control level.

Each sample was analysed in duplicate. The variability between duplicates was between 0 and 4.8%. To investigate inter-plate variability, between five and 10 randomly selected samples were repeated on different test plates and serial dilutions of the positive control cDNA were also performed. Inter-plate reproducibility was between 3.7 and 15.5%.

#### Positive control

Previous work undertaken by our group has identified the pancreatic cancer cell line MIA PaCa2 to consistently produce PIF-CP mRNA. This cell line was, therefore, used as positive control for real-time PCR analysis. Total RNA was collected from cultured cells by the Trizol extraction method (Invitrogen, Renfrew, UK) before undergoing reverse transcription as described above.

### Acute-phase proteins

C-reactive protein was determined using an immunoturbidimetric assay (Abbott TDX, Abbott Laboratories, Maidenhead, UK). A level above 10 mg l^−1^ represents the presence of an acute-phase response. The remaining acute-phase proteins (transferrin, haptoglobin, and *α*1-antichymotrypsin) were determined by enzyme-linked immunosorbent assay as described previously (Wigmore *et al*, 2002).

### Statistical analysis

Differences in levels of PIF-CP mRNA expression between different tissue types and PIF urinary expression were analysed by the Mann–Whitney *U*-test. Survival differences were tested by the log-rank test. Comparisons between tumour tissue PIF-CP gene expression and benign tissue expression were tested by linear regression analysis following natural logarithmic transformation of the data. Stage was analysed by the *χ*^2^ test. Probabilities ⩽0.05 were considered significant.

## RESULTS

### Study patients

Patient demographics are shown in Table 1. Forty-six patients were recruited to the study, the median age was 65 years (inter-quartile range 58–75) and 32 (70%) of the patients were men. The primary tumour sites were oesophageal (*n*=22, 48%), gastric (*n*=15, 33%), and those arising from the gastro-oesophageal junction (*n*=9, 20%). Histological confirmation of disease was obtained in all cases and the predominant histological subtype was adenocarcinoma (94%). All patients underwent surgical resection and 11 (24%) of these received preoperative chemotherapy. At the end of the study, 11 (24%) patients had died.

All the control patients were considered healthy and all were weight stable. These patients underwent endoscopy as an elective investigation and in all instances the result of the procedure was normal, including both macroscopic and microscopic assessment. Helicobacter pylori was not detected in any of these tissue samples.

### Expression of PIF-CP mRNA in tissues

#### Healthy controls

**Figure 1:**
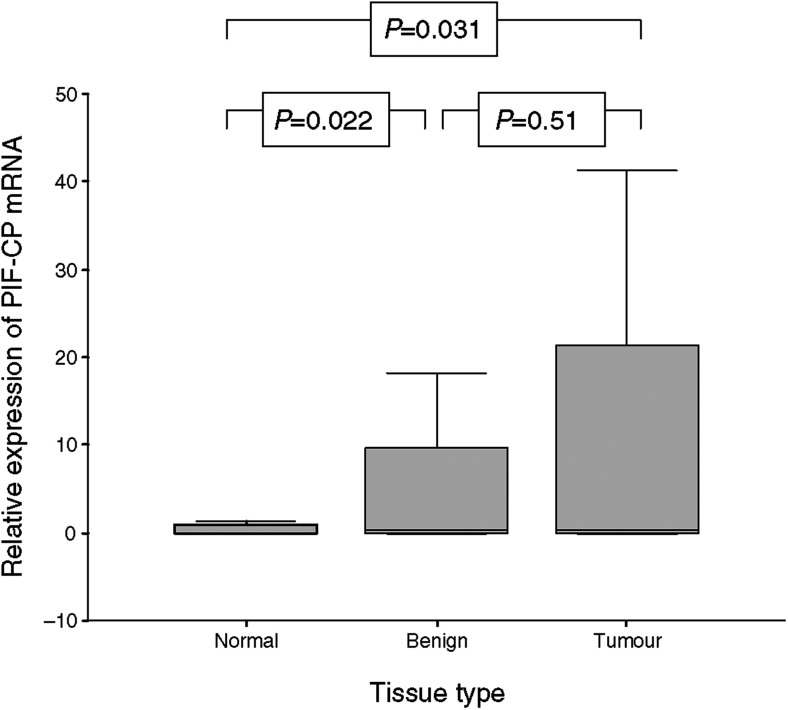
A comparison of relative expression of PIF-CP mRNA in tissue from healthy controls (normal), tumour tissue, and benign tissue collected from cancer patients (benign). The lines represent the median value, bars=inter-quartile range, error bars=extreme values (Mann–Whitney *U*-test).

mRNA of PIF-CP was detected in gastro-oesophageal biopsy tissue from four (36%) healthy controls. Of these positive tissue samples, three were gastric biopsies, and one was oesophageal tissue. Although PIF-CP mRNA levels were detectable, the levels of expression were significantly lower when compared with levels of expression in tumour tissue (*P*=0.031, Mann–Whitney *U*-test) and adjacent benign tissue (*P*=0.022) taken from cancer patients (Figure 1).

#### Study patients

**Figure 2:**
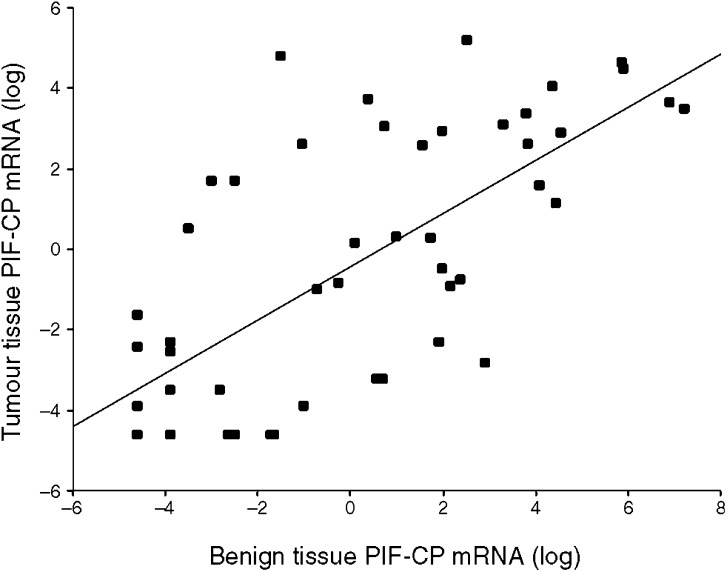
Correlation between paired tumour tissue PIF-CP mRNA concentrations and benign tissue PIF-CP mRNA concentrations (*P*<0.0001, *r*=0.73; linear regression). All values underwent natural logarithmic transformation.

PIF-CP mRNA was detected in 27 (59%) tumour samples and 31 (67%) adjacent benign tissue samples. In 24 (52%) patients, PIF mRNA was detected in both the tumour tissue and adjacent benign tissue collected from the same patient (Table 2). There was a strong correlation between paired mRNA concentrations in tumour tissue and mRNA concentrations in adjacent benign tissue (*P*<0.0001, *r*=0.73; linear regression) (Figure 2). However, there was no difference in the level of gene expression between tumour tissue and adjacent benign tissue from the cancer patients (*P*=0.51, Mann–Whitney *U*-test) (Figure 1). In 12 (26%) patients, PIF-CP mRNA was not detected in either the tumour or adjacent benign tissue. For the remaining 10 patients, PIF-CP mRNA was detected in either tumour tissue only (*n*=3) or adjacent benign tissue only (*n*=7). There was no difference in the level of gene expression between patients who received preoperative chemotherapy and those who did not (tumour tissue, *P*=0.58; adjacent benign tissue, *P*=0.72: Mann–Whitney *U*-test).

### Tissue PIF-CP mRNA expression and nutritional status

Tumour tissue PIF-CP mRNA did not correlate with weight loss (*P*=0.37; linear regression), MAC (*P*=0.10), triceps skinfold thickness (*P*=0.37), or AMC (*P*=0.14). Similarly, benign tissue mRNA concentrations did not correlate with weight loss (*P*=0.84) or any anthropometric measurements. Patients in whom PIF-CP mRNA was measurable in both tumour and adjacent benign tissues did not have adverse nutritional status when compared with patients without detectable PIF-CP mRNA in either tissue type (Table 3). There remained no correlation when the data were analysed according to dysphagia scores (data not shown).

### Tissue PIF-CP mRNA expression and systemic inflammation

Nine (20%) patients had an elevated serum CRP (>10 mg l^−1^), which may be used as a surrogate marker of systemic inflammation. There was no association between levels of PIF-CP mRNA in either tumour tissue (*P*=0.89) or adjacent benign tissue (*P*=0.81) and elevated serum acute-phase protein concentrations (Mann–Whitney *U*-test).

### Tissue PIF-CP mRNA expression and prognosis

PIF-CP mRNA in either tumour tissue (*P*=0.64) or benign tissue (*P*=0.51) was not associated with adverse prognosis (Mann–Whitney *U*-test). Similarly, those patients with detectable mRNA in both tumour and benign tissue did not have adverse survival compared with patients with no detectable PIF-CP gene in either tissue type (*P*=0.79; *χ*^2^ analysis).

We found no association between mRNA levels and age, sex, tumour position, histology, degree of differentiation, or stage (data not shown).

## DISCUSSION

Previous studies have suggested that PIF-CP mRNA is absent or expressed at only minimal levels in normal human tissues and found at significantly elevated levels in some tumours and various cancer cell lines. However, we have detected PIF-CP mRNA in benign tissue in the majority of patients with gastro-oesophageal cancer, and these levels are equivalent to levels detected within adjacent tumour tissues. In addition, we detected PIF-CP mRNA in biopsy tissue from healthy controls, although at significantly lower levels of expression. In a previous study, PIF-CP (HCAP) mRNA appeared to be limited to cancer cells and their metastases (Wang *et al*, 2003). Our data suggest that PIF-CP gene expression is not limited to neoplastic tissue and that the PIF-CP gene may also be active in normal tissues. Furthermore, the level of gene expression appears to be similar in tumour tissue and adjacent benign tissue. Therefore, in some individuals with cancer, there may be upregulation of the PIF-CP gene not only in tumour tissue but also within the whole organ or even in distant tissues.

The relevance of PIF-CP gene upregulation remains obscure. Although PIF-CP has been identified as a putative cell survival factor we found no association between gene expression and prognosis, stage, or grade of tumour. Therefore, the present study is unable to confirm a tumour survival-promoting role for this gene in our study. Mice implanted with a tumour carrying the PIF-CP gene demonstrated increased rate of tumour growth compared with controls (Monitto *et al*, 2004). The present study did not evaluate tumour volume, but we did not identify any differences in tumour stage or grade between patients with elevated mRNA levels and those with undetectable mRNA levels.

Tissue PIF-CP mRNA expression was not associated with weight loss or any adverse nutritional variable. Previously, we have found an association between weight loss and urinary PIF (Todorov *et al*, 1996; Cariuk *et al*, 1997; Wigmore *et al*, 2000). In those studies, we detected the glycosylated PIF protein, whereas in this study, we have measured mRNA for the core peptide and so this finding is perhaps unsurprising. Only a small fraction of the total peptide core would be expected to be glycosylated, and as PIF is only present at 1 part in 10^8^ of the total protein, expression of the peptide core would not be expected to be rate limiting for PIF formation, rather the expression of the glycosidases, their rate of catalysis, and substrate availability (Todorov *et al*, 1996). Whether variation in glycosylation of PIF occurs and whether this influences biological activity is unknown. Antibodies raised against the core peptide do not reliably detect urinary PIF.

Systemic inflammation was identified in 20% of patients and was not associated with PIF core tissue mRNA concentrations. *In vitro* work has demonstrated that hepatocytes produce increased levels of IL-6, IL-8, and CRP in response to PIF stimulation (Watchorn *et al*, 2001). This is thought to be mediated through transcription factors NF-κB and STAT3. Proteolysis-inducing factor may play only a minor role in the complex interaction between proinflammatory mediators and a direct relationship between PIF and acute-phase proteins may not be identified outwith the *in vitro* cell model.

It has been suggested that PIF is an important protein during embryological development and becomes quiescent during adult life (Watchorn *et al*, 2001). Neoplastic transformation would then allow the re-expression of the gene in adulthood (similar to carcinoembryonic antigen). We have found PIF-CP mRNA expression in normal healthy tissue albeit at reduced levels compared with tumour tissue and benign tissue from cancer patients and so this explanation is perhaps implausible for PIF-CP but may be important in re-expression of the glycosyltransferases. It is also important to consider that real-time PCR is an exquisitely sensitive technique and that what we are detecting in some patients, although elevated, may have little or no functional significance as it may not be translated into protein.

It is possible that the gene for the PIF-CP may confer a survival advantage to a tumour, promoting tumour growth and spread, although this study was unable to confirm this. Alternatively, the PIF-CP gene may be expressed to some degree in all tissue types and its diverse functions are due to post-transcriptional and post-translational modifications. The high degree of glycosylation of the PIF molecule may be due to aberrant glycosylation by tumour cells and this glycosylation may confer the proteolytic activity associated with PIF (Todorov *et al*, 1997). This aberrantly processed molecule could not be distinguished from any conventional versions by determination of the mRNA expression.

PIF-CP mRNA is expressed in healthy tissue and is found at significantly elevated levels within tumour tissue and adjacent benign tissue. However, measuring mRNA expression is unreliable as an indicator of glycosylated PIF protein expression and therefore has limited value both as a diagnostic and prognostic test. Attention needs to be directed at developing robust and reproducible quantitative assessments of glycosylated PIF protein. Such tests need to be able to identify PIF in complex matrices such as plasma or urine and must be able to identify glycosylation variants of the moiety. Similarly, such tests need to distinguish between different proteins carrying identical glycosylation motifs.

## Figures and Tables

**Table 1:** Patient demographics (*n*=46)

	**Number (%)**
Age (years)	65 (58–75)
	
*Sex*	
Male	32 (70)
Female	14 (30)
	
*Tumour site*	
Oesophageal	22 (48)
Oesophago-gastric junction	9 (20)
Gastric	15 (33)
	
*Histology*	
Adenocarcinoma	43 (94)
Squamous cell carcinoma	3 (6)
	
*Grade*	
Well differentiated	4 (9)
Moderately differentiated	20 (44)
Poorly differentiated	22 (48)
	
*UICC stage*	
1	14 (30)
2	12 (26)
3	15 (33)
4	5 (11)
	
*Treatment undertaken*	
Oesophagectomy alone	20 (43)
Gastrectomy alone	15 (33)
Preoperative chemotherapy followed by surgery	11 (24)
	
*Status*	
Alive	35 (76)
Dead	11 (24)

Values are median (inter-quartile range). UICC=International Union Against Cancer.

**Table 2:** Tissue expression of PIF-CP mRNA in paired tumour tissue and benign tissue

Paired tumour tissue and benign tissue expression	Benign tissue expression only
24 (52%)	7 (15%)
Tumour tissue expression only	Neither tumour tissue nor benign tissue expression
3 (7%)	12 (26%)

PIF-CP=proteolysis-inducing factor core peptide.

**Table 3:** Tissue PIF-CP mRNA expression and nutritional variables

	**PIF-CP mRNA detected in both tumour tissue and benign tissue (*n*=24)**	**PIF-CP mRNA not detectable in either tumour tissue or benign tissue (*n*=12)**	***P* value**
Weight loss (%)	3.3 (0.4–9.5)	5.3 (0–12.2)	0.93
MAC (percentile group)	10–25 (5–50)	10–25 (5–50)	0.96
Triceps (percentile group)	25–50 (10–50)	25–50 (25–50)	0.37
AMC (percentile group)	10–25 (5–50)	25–50 (5–50)	0.96

Nutritional variables were similar between patients in whom PIF-CP mRNA was measurable in both tumour tissue and benign tissue and patients who had no detectable mRNA (Mann–Whitney *U*-test). Absolute values were normalised into percentile groups before analysis.

Values are median (inter-quartile range). PIF-CP=proteolysis-inducing factor core peptide, MAC=mid-arm circumference, triceps=triceps skinfold thickness, AMC=arm muscle circumference.
